# Erythroderma: a prospective study of 309 patients followed for 12 years in a tertiary center

**DOI:** 10.1038/s41598-020-66040-7

**Published:** 2020-06-17

**Authors:** Denis Miyashiro, José Antonio Sanches

**Affiliations:** Division of Clinical Dermatology, Hospital das Clínicas, University of São Paulo Medical School, São Paulo, Brazil

**Keywords:** Skin cancer, Skin manifestations

## Abstract

Erythroderma is characterized by erythema and scaling affecting more than 80% of the body surface area. It is potentially life-threatening, and diagnosis of the underlying disease is a challenge. Despite laboratory improvements, many cases remain idiopathic. We aimed to analyze clinical and laboratory findings of 309 erythrodermic patients to find clues to the etiologic diagnosis. We performed a prospective study at the University of São Paulo Medical School, from 2007 to 2018, with patients with acquired erythroderma. Clinical, laboratory, histology, and molecular biology data were collected. The median age at diagnosis was 57 years, with a male-to-female ratio of 2.2. Eczema was the most frequent etiology (20.7%), followed by psoriasis (16.8%), Sézary syndrome (12.3%), drug eruption (12.3%), atopic dermatitis (8.7%), and mycosis fungoides (5.5%). Other diagnoses (6.8%) included pemphigus foliaceous, paraneoplastic erythroderma, adult T-cell leukemia/lymphoma, dermatomyositis, pityriasis rubra pilaris, lichen planus, bullous pemphigoid, and leprosy. In 52 patients (16.8%), it was not possible to elucidate erythroderma etiology. Atopic dermatitis developed erythroderma at an earlier age (median 25 years; *P* = 0.0001). Acute onset was associated with drug reactions and atopic dermatitis (median time from erythroderma to diagnosis of 1 and 1.5 months, respectively; *P* = 0.0001). Higher immunoglobulin E levels were observed in atopic dermatitis (median 24,600 U/L; *P* = 0.0001). Histopathology was helpful and was consistent with the final diagnosis in 72.4%. Monoclonal T-cell proliferation in the skin was observed in mycosis fungoides (33.3%) and Sézary syndrome (90.9%). At the last assessment, 211 patients (69.3%) were alive with disease, 65 (21.7%) were alive without disease, and 27 (9.1%) died with active disease. Erythroderma is a challenging syndrome with a difficult diagnostic approach. Younger age and higher immunoglobulin E levels are associated with atopic dermatitis; acute onset is observed in drug eruptions and atopic dermatitis. Histopathology and molecular biology tests are essential tools in the investigation of erythroderma.

## Introduction

Erythroderma, or exfoliative dermatitis, is an inflammatory disorder characterized by erythema and scaling affecting more than 80% of the body surface area^1^. It decreases the quality of life and is a potentially fatal disease. Hospitalization is usually necessary for initial evaluation and treatment. Several skin disorders may culminate with erythroderma: exacerbation of previous dermatoses (*e.g*., psoriasis, eczema, atopic dermatitis, pityriasis rubra pilaris, pemphigus foliaceous), drug eruption, and cutaneous lymphomas (mycosis fungoides, Sézary syndrome, adult T-cell leukemia/lymphoma). The challenge in these patients is represented by the identification of the etiological agents or conditions, which is relevant in clinical management and prognosis. Despite extensive investigation, some patients are classified as idiopathic erythroderma^2^.

Several studies reported the frequencies of different etiologies of erythroderma. The association between clinical findings and the etiology is reported, but results are heterogeneous, and no consistent association has been confirmed. Studies reporting laboratory alterations are scarce, and histopathology patterns of these diseases often resemble the classical form of the disorder (non-erythrodermic). However, frequently, non-specific histopathologic alterations cause difficulties in the diagnosis^3^.

We aim to analyze clinical, laboratory, histopathological, and molecular findings of erythrodermic patients followed prospectively in a tertiary center in Brazil between January 2007 and December 2018, to find clues to the etiology, and to propose a diagnostic approach to facilitate the management of these patients.

## Methods

We performed a study with 309 patients at the Division of Clinical Dermatology, Hospital das Clínicas, University of São Paulo Medical School, from January 2007 to December 2018. All patients had acquired erythroderma, with erythema and scaling affecting more than 80% of the body surface area for more than one month. Congenital erythroderma was excluded, and patients with generalized poikiloderma were not considered as erythroderma. From 2007 to 2013, data from 180 patients were collected prospectively by a single researcher (DM), and, according to these findings, a detailed clinical and laboratory protocol was developed (Table 1). From 2014 to 2018, a prospective analysis based on this protocol was performed on 129 patients. For all clinical data described and analyzed in this manuscript, we considered only the patients evaluated after the establishment of the detailed protocol (129 patients, from 2014 to 2018). Since laboratory results do not depend on dermatological information, these data were analyzed in the whole cohort (309 patients).

**Table 1:** Data collected at initial evaluation.

Demographic data
Age
Sex
Ethnicity
**Clinical data**
Time of onset of erythroderma to the first evaluation
Previous cutaneous diseases
Exposition to new medications or possible allergens
Comorbidities
Weight loss
Pruritus
Non-scarring diffuse alopecia
Palmo-plantar keratoderma
Ungueal alterations
Ectropion
Lower limb edema
Mucosal lesions
Achromic vitiligo-like lesions
Spared areas
Lymph node enlargement
Hepatosplenomegaly
Temperature
Heart rate
Respiratory rate
Blood pressure
**Laboratory tests**
Complete blood count (leukocytosis if ≥ 11,000/mm^3^; lymphocytosis if ≥ 3,400/mm^3^; eosinophilia if ≥ 500/mm^3^)
Immunophenotyping of lymphocytes by flow cytometry^8^
Sézary cell count in peripheral blood smear^68^
T-cell receptor (TCR) gene rearrangement analysis by PCR in the blood^9^
Lactate dehydrogenase
Protein electrophoresis
Total IgE
HIV serology
HTLV 1/2 serology^69^
Three simultaneous skin biopsies to histology
One skin biopsy to TCR gene rearrangement analysis
Computed tomography (CT) of thorax, abdomen, and pelvis
Lymph-node excisional biopsy if ≥ 1.5 cm, or if abnormal characteristics are found (firm, irregular, clustered, or fixed)

We classified patients into seven subgroups: psoriasis, atopic dermatitis (AD), eczema, drug-induced erythroderma, mycosis fungoides (MF), Sézary syndrome (SS), miscellaneous, and idiopathic cases (when patients did not fulfill criteria for any of the previous diagnoses at first evaluation and follow-up).

The final diagnosis was based on clinical and pathological characteristics, laboratory findings, and follow-up data. Diagnosis of SS was based on the World Health Organization - European Organization for Research and Treatment of Cancer (WHO-EORTC) criteria^4^. Manual Sézary cell count is difficult to perform and is a subjective test. Thus, many European and American centers are not performing this test anymore, with a preference for immunophenotyping of lymphocytes by flow cytometry^5^. In our study, some manual Sézary cell count did not provide the absolute number of cells and, consequently, we did not consider these cases for analysis. In those cases, SS diagnosis was based on the other criteria described on the WHO-EORTC consensus^4,6^. The methodologies used for manual Sézary cell count, immunophenotyping of lymphocytes by flow cytometry, and T-cell clonality were previously described^7–9^. Alterations in immunophenotyping of lymphocytes by flow cytometry included CD4/CD8 ≥ 2.8; CD4 + CD7- ≥ 40%; CD4 + CD26- ≥ 30%. We further divided the patients with CD4/CD8 ≥ 2.8 into two groups. One with CD4/CD8 between 2.8 and 10, and one with CD4/CD8 ≥ 10, because the last is a criterion for SS.

For skin histopathology, we considered the impression of the dermatopathologist, if the conclusion was consistent with the final diagnosis. Also, we collected data on the presence of spongiosis and exocytosis of lymphocytes. These findings are commonly observed in eczema, and cutaneous T-cell lymphomas (CTCL), respectively, and the associations of those characteristics in the biopsy of erythrodermic patients with different etiologies were analyzed.

Statistical analyses were performed with STATA version 13 (STATA Corp., Texas, United States). Shapiro-Wilk test of normality showed that all quantitative data were non-parametric. Thus, the median and interquartile range (IQR), as well as Kruskal-Wallis with Dunn post-test, were used to describe data and associations. Qualitative data are shown as frequency and percentages, and analyses were performed with chi-squared or Fisher’s exact tests. Statistical significance was considered when *P* ≤ 0.05.

The study was approved by the local ethics committee in the University of São Paulo Medical School (CAPPesq – Comissão de Ética para Análise de Projetos de Pesquisa) with internal code 13494 and followed the principles of the Declaration of Helsinki. Informed consent from all participants of the study was obtained.

## Results

### Demographic and clinical findings

Among 309 patients, there were 213 males (68.9%) and 96 females (31.1%), with a male-to-female ratio of 2.2. The median age at diagnosis of erythroderma was 57 years. The etiologies and demographic data are summarized in Tables 2 and 3.

**Table 2:** Etiologies.

Diagnosis	N (%)
Psoriasis	52/309 (16.8)
Eczema	64/309 (20.7)
AD	27/309 (8.7)
Drug-induced erythroderma	38/309 (12.3)
SS	38/309 (12.3)
MF	17/309 (5.5)
Miscellaneous^**†**^	21/309 (6.8)
Idiopathic	52/309 (16.8)

†Pemphigus foliaceous (7/21–33.3%), paraneoplastic erythroderma (4/21–19.0%), adult T-cell leukemia/lymphoma (3/21–14.3%), dermatomyositis (2/21–9.5%), pityriasis rubra pilaris (2/21–9.5%), lichen planus (1/21–4.8%), bullous pemphigoid (1/21–4.8%), leprosy (1/21–4.8%).

**Table 3:** Demographic data.

Diagnosis	Male: Female (ratio)	Median age, years (IQR^†^)	Ethnicity (%)	Median time from the onset of erythroderma to the first evaluation, months (IQR)	Median time of follow-up, months (IQR)
Psoriasis	36:16 (2.2)	48 (36.5–57)	White: 36 (69.2)	2.0 (1.0–5.0)	51.6 (15.5–84.9)
			Black: 7 (13.5)		
			Brown: 9 (17.3)		
			Yellow: 0		
Eczema	43:21 (2.0)	60.5 (53–71.5)	White: 33 (51.6)	4.0 (2.0–8.0)	16.2 (4.1–51.9)
			Black: 16 (25.0)		
			Brown: 14 (21.9)		
			Yellow: 1 (1.6)		
AD	19:8 (2.4)	25 (19–32)*	White: 13 (48.1)	1.5 (1.0–7.0)*	53.7 (9.9–107.4)
			Black: 5 (18.5)		
			Brown: 8 (29.6)		
			Yellow: 1 (3.7)		
Drug-induced	23:15 (1.5)	62.5 (47–71)	White: 32 (84.2)	1.0 (1.0–3.0)*	12.7 (3.9–29.7)
			Black: 3 (7.9)		
			Brown: 3 (7.9)		
			Yellow: 0		
SS	22:16 (1.4)	62.5 (54–70)	White: 26 (68.4)	11.0 (4.0–24.3)	14.1 (7.3–33.9)
			Black: 5 (13.1)		
			Brown: 7 (18.4)		
			Yellow: 0		
MF	13:4 (3.2)	58 (51–69)	White: 6 (35.3)	12.0 (3.2–24.3)	28.0 (14.4–89.2)
			Black: 1 (5.9)		
			Brown: 9 (52.9)		
			Yellow: 1 (5.9)		
Miscellaneous	14:7 (2.0)	56 (45–63)	White: 15 (71.4)	6.0 (2.0–12.0)	18.0 (6.2–31.7)
			Black: 1 (4.8)		
			Brown: 5 (23.8)		
			Yellow: 0		
Idiopathic	43:9 (4.8)	68 (57–77)	White: 23 (44.2)	4.5 (3.0–10.0)	14.4 (5.4–31.9)
			Black: 11 (21.1)		
			Brown: 14 (26.9)		
			Yellow: 4 (7.7)		
Total	213:96 (2.2)	57 (45–70)	White: 184 (59.5)	4.0 (1.0–10.0)	18.5 (6.5–54.0)
			Black: 49 (15.9)		
			Brown: 69 (22.3)		
			Yellow: 7 (2.3)		

^†^IQR: interquartile range.

*P = 0.0001.

Among the 129 patients evaluated with the detailed protocol, 49/129 (38%) had a history of previous skin disease: all patients with erythrodermic AD (13/13) had a history of previous AD, and 14/18 (77.8%) patients with erythrodermic psoriasis had a history of psoriasis vulgaris. Four patients with SS (4/19, 21%) had a history of previous skin disease: two had eczema, one had reticular erythematous mucinosis, and one had MF. Ten (10/27, 37%) patients with eczema had a history of previous eczematous disorder; two (2/9, 22.2%) patients with drug-induced erythroderma had a history of previous eczema; one patient with erythrodermic dermatomyositis had previous diagnosis of psoriasis; and five (5/25, 20%) patients with idiopathic erythroderma had previous history of eczematous reactions. The causative agents in drug-induced erythroderma were identified in 21 patients (21/38, 55.3%): anticonvulsants (13/21, 61.9%), antihypertensives (4/21, 19.0%), anti-inflammatory (3/21, 14.3%), and oral hypoglycemic drugs (1/21, 4.8%). The other patients with drug-induced erythroderma (17/38, 44.7%) had multiple agents involved, and a single causative agent could not be reached.

Detailed clinical findings are described in Table 4. Palmoplantar keratoderma, from mild keratosis and scaling to severe thickening, was observed in 90/128 (70.3%) patients, and it was more common in psoriasis (16/18, 88.9%), SS (16/19, 84.2%), and in idiopathic cases (22/25, 88.0%) (*P* = 0.005).

Nail alterations were observed in 68/129 (52.7%) patients and were more frequent in psoriasis (15/18, 83.3%) and SS (15/19, 78.9%) (*P* < 0.0001). Patients with psoriasis had subungual hyperkeratosis (8/18, 44.4%), pitting (6/18, 33.3%), onycholysis (3/18, 16.7%), and oil drop sign (2/18, 11.1%). Patients with SS had subungual hyperkeratosis (10/19, 52.6%), onycholysis (6/19, 31.6%), and shiny nails due to severe pruritus (2/19, 10.5%).

Sixty-one patients (61/128, 47.7%) had erythroderma with areas of healthy skin. It was more common in eczema (19/27, 70.4%) and AD (9/13, 69.2%) (*P* = 0.035), and the central area of the face was the most common spared area (51/61, 83.6%).

Other findings are described in Table 4, and differences between the etiologies did not reach statistical significance.

**Table 4:** Clinical findings.

	Psoriasis	Eczema	AD	Drug reaction	SS	MF	Miscellaneous	Idiopathic	Total
Alopecia (%)	4 (22.2)	6 (22.2)	3 (23.1)	5 (55.6)	10 (52.6)	4 (66.7)	5 (41.7)	9 (36.0)	46 (35.7)
PPK* (%)	16 (88.9)	14 (51.8)	5 (38.5)	7 (77.8)	16 (84.2)	3 (60.0)	7 (58.3)	22 (88.0)	90 (70.3)
Fissures (%)	7 (38.9)	7 (25.9)	3 (23.1)	3 (33.3)	11 (57.9)	1 (16.7)	5 (41.7)	11 (44.0)	48 (37.2)
Ungueal alterations** (%)	15 (83.3)	9 (33.3)	2 (15.4)	3 (33.3)	15 (78.9)	3 (50.0)	5 (41.7)	16 (64.0)	68 (52.7)
Weight loss (%)	9 (50.0)	8 (29.6)	5 (38.5)	7 (77.8)	10 (52.6)	4 (66.7)	7 (58.3)	14 (56.0)	64 (49.6)
Pruritus (%)	18 (100)	27 (100)	13 (100)	8 (88.9)	19 (100)	6 (100)	11 (91.7)	25 (100)	127 (98.4)
Lower limb edema (%)	14 (77.8)	20 (74.1)	4 (30.8)	6 (66,67)	11 (57.9)	3 (50.0)	8 (66.7)	14 (56.0)	80 (62.0)
Ectropion (%)	5 (27.8)	4 (14.8)	2 (15.4)	3 (33,33)	6 (31.6)	1 (16.7)	5 (41.7)	11 (44.0)	37 (28.7)
Mucosal lesions (%)	2 (11.1)	0	0	1 (11.1)	0	0	1 (8.3)	3 (12.0)	7 (5.4)
Vitiligoid lesions (%)	3 (16.7)	5 (18.5)	2 (15.4)	4 (44.4)	3 (15.8)	0	0	6 (24.0)	23 (17.8)
Areas of normal skin^†^ (%)	7 (41.2)	19 (70.4)	9 (69.2)	2 (22.2)	6 (31.6)	1 (16.7)	5 (41.7)	12 (48.0)	61 (47.7)
Lymph node enlargement (%)	6 (33.3)	14 (51.8)	9 (69.2)	4 (44.4)	12 (63.2)	4 (66.7)	4 (33.3)	16 (64.0)	69 (53.5)
Hepatosplenomegaly (%)	0	0	0	0	1 (5.26)	0	1 (8.3)	1 (4.0)	3 (2.3)
Fever (%)	4 (22.2)	1 (3.7)	0	2 (22.2)	0	0	2 (16.7)	0	9 (6.7)
Tachycardia (%)	5 (27.8)	2 (7.4)	2 (15.4)	1 (11.1)	1 (5.26)	0	4 (33.3)	0	15 (11.6)
Hypotension (%)	1 (5.6)	0	0	0	0	0	1 (8.3)	0	2 (1.5)
Taquipnea (%)	1 (5.6)	0	1 (7.7)	0	1 (5.26)	0	4 (33.3)	3 (12.0)	10 (7.7)

*P = 0.005; **P = 0.0001; ^**†**^*P* = 0.035.

## Histopathology findings

Histopathology findings are described in Table 5. Biopsies of the skin were consistent with the final diagnosis in 199/275 patients (72.4%) and were non-specific in 39/275 (27.6%). Spongiosis was observed in 191/272 patients (69.4%), and it was more common in eczematous disorders (100% in eczema and AD patients, p < 0.001). Exocytosis of lymphocytes was observed in 126/275 patients (45.8%) and was observed more frequently in SS (29/38, 76.3%), MF (11/16, 68.7%), drug-induced erythroderma (22/34, 64.7%), and idiopathic cases (31/52, 59.6%) (*P* < 0.001).

Lymph node biopsy was performed in 60/309 patients (19.4%). Four of the nine biopsies in MF (44.4%) and 6/21 biopsies in SS (28.6%) showed infiltration of the lymph node by neoplastic cells, but the difference between these groups did not reach statistical significance (*P* = 0.384).

Bone marrow biopsy was performed in eight patients (8/303, 2.6%): two with drug-induced erythroderma due to hypereosinophilia, three with ATLL, one with MF and two with SS due to cytopenias. Infiltration by neoplastic cells was observed in two patients with SS and two with ATLL; the patients with drug-induced erythroderma and MF had normal bone marrow tissue.

## Imaging findings

Imaging studies are described in Table 5. Lymph node enlargement was observed in 104/185 patients (56.2%) and was more frequent in SS (25/33, 75.8%), MF (13/15, 86.7%), and drug-induced erythroderma (10/14, 71.4%) (*P* = 0.004). The most frequently involved sites were axillae (79/185, 42.7%) and inguinal regions (72/185, 38.9%). Mediastinal and intraabdominal lymph node enlargement was observed in 35/185 (18.9%) and 23/185 (12.4%) patients, respectively. Among patients with mediastinal lymph node enlargement, 8 (22.9%) had SS, 6 (17.1%) drug eruption, 5 (14.3%) psoriasis, 5 (14.3%) idiopathic erythroderma, 4 (11.4%) eczema, 3 (8.6%) MF, 1 (2.9%) AD, 3 (8.6%) other diagnosis (paraneoplastic erythroderma, ATLL, dermatomyositis) (p = 0.342). Among patients with intraabdominal lymph node enlargement, 4 (17.4%) had SS, 4 (17.4%) had psoriasis, 3 (13.0%) had MF, 3 (13.0%) had eczema, 3 (13.0%) had drug eruption, 3 (13.0%) had other diagnoses (2 ATLL, 1 dermatomyositis), and 3 (13.0%) had idiopathic erythroderma (*P* = 0.551).

## Laboratory findings

Laboratory findings are described in Table 5. The median leukocyte count was 9,600 cells/mm^3^, with higher values in SS (13,575 cells/mm^3^; *P* = 0.0002). The median lymphocyte count was 1,800 cells/mm^3^, with higher values in SS (4,400 cells/mm^3^; *P* = 0.0001). Sézary cells in peripheral blood were detected in 32/176 patients (18.2%) but were not always quantified. It was present in 15/25 patients (60.0%) with SS, 7/16 (43.7%) with MF, 2/11 (18.2%) in miscellaneous group, 4/43 (9.3%) with idiopathic, 3/35 (8.6%) with eczema, and 1/19 (5.3%) with drug-induced erythroderma. Sézary cells were not observed in AD or psoriasis.

The median eosinophil count was 565 cells/mm^3^, with higher values in AD (1,700 cells/mm^3^) and idiopathic cases (1,035 cells/mm^3^) (*P* = 0.0001). Total IgE was increased in 143/170 patients (84.1%). Median IgE was 975.6 UI/mL (normal range<100 UI/mL), but increased values were more pronounced in AD, with median value of 24,600 UI/mL (*P* = 0.0001).

Elevated LDH was observed in 181/281 patients (64.4%). Increased values were less frequent in psoriasis (12/48, 25.0%) (*P* < 0.001). Hypoalbuminemia (albumin <3.4 g/dL) was observed in 93/238 (35.6%) patients. HTLV-1 infection was observed in 7/238 patients (2.9%): three with ATLL, two idiopathic erythroderma, one with drug-induced erythroderma, and one with eczema. Except for ATLL, the other HTLV-1-positive patients had no neoplastic cells on the blood or any tissue. HIV was positive in 6/276 (2.2%) patients, one with eczema (1.9%), two with MF (11.8%), and three with psoriasis (6.2%). HIV was more frequent in MF and psoriasis groups (*P* = 0.057).

### Flow cytometry analysis

Alterations in flow cytometry of peripheral blood are described in Table 5. The CD4/CD8 ≥ 2.8; CD4 + CD7- ≥ 40%; or CD4 + CD26- ≥ 30% were observed in 65/218 patients (29.8%). As expected, flow cytometry alterations were observed in all patients with SS (38/38, 100%) (*P* < 0.001), once it is a diagnostic criteria for SS. Alterations were also observed in 4/12 patients (33.3%) with AD; 3/26 (11.5%) with drug-induced erythroderma; 12/50 (24.0%) with idiopathic erythroderma; 3/17 (17.6%) with MF; 2/13 (15.4%) in the miscellaneous group, one patient with chronic ATLL (CD4/CD8 of 11.4, CD4 + CD7- of 41.0%), one with paraneoplastic erythroderma due to lung adenocarcinoma (CD4/CD8 of 5.1, CD4 + CD7- of 55.0%); 3/39 (7.7%) with eczema; and no patient with psoriasis.

**Table 5:** Histopathology, imaging, and laboratory alterations.

	Psoriasis	Eczema	AD	Drug reaction	SS	MF	Miscellaneous	Idiopathic	Total
Skin biopsy consistent with de final diagnosis (%)*	44/44 (100)	50/52 (96.1)	16/17 (94.1)	29/34 (85.3)	28/38 (73.7)	16/17 (94.1)	16/21 (76.2)	0/52 (0)	199/275 (72.4)
Spongiosis (%)*	15/44 (34.1)	52/52 (100)	17/17 (100)	24/34 (70.6)	20/38 (52.6)	7/17 (41.2)	9/21 (42.9)	47/52 (90.4)	191/275 (69.4)
Exocytosis of lymphocytes (%)*	4/44 (9.1)	21/53 (39.6)	4/17 (23.5)	22/34 (64.7)	29/38 (76.3)	11/16 (68.7)	4/21 (19.0)	31/52 (59.6)	126/275 (45.8)
Lymph node biopsy	1 DL (100%)	0	0	0	15 DL (71.4%) 6 N2/3 (28.6%)	5 DL (55.6%) 4 N2/3 (44.4%)	0	1 DL (100%)	22 DL (68.7%) 10 N2/3 (31.3%)
Lymph node enlargement on CT scan (%)**	11/25 (44.0)	13/36 (36.1)	5/11 (45.4)	10/14 (71.4)	25/33 (75.8)	13/15 (87.7)	7/15 (46.7)	20/36 (55.6)	104/185 (56.2)
Median leukocytes, cells/mm^3^ (IQR)^†^	9,130 (7,340–11,615)	9,260 (7,070–11,595)	9,700 (7,590–13,430)	10,500 (8,000–13,050)	13,575 (8,570–22,180)	7,320 (6,490–8,720)	10,470 (9,010–16,250)	9,555 (7,860–12,280)	9,600 (7,470–12,800)
Median lymphocytes, cells/mm^3^ (IQR)^††^	1,870 (1,335–2,300)	1,615 (1,200–2,145)	1,800 (1,310–2,370)	1,600 (1,100–2,300)	4,400 (2,100–9,200)	1,700 (1,100–2,000)	2,150 (1,500–3,030)	1,585 (1,185–2,100)	1,800 (1,300–2,530)
Median eosinophils, cells/mm^3^ (IQR) ^††^	345 (200–600)	640 (215–1,145)	1,700 (500–2,900)	500 (200–2,000)	265 (140–890)	640 (200–800)	390 (100–1,340)	1,035 (400–1,550)	565 (200–1,270)
Median total IgE, U/mL (IQR)^††^	782 (138.2–3,680)	1,285.5 (442–2,430)	24,600 (7,370–38,250)	99.5 (44–254)	309 (78–1,720)	731 (551–1,740)	1,103 (643–5,616)	439 (170–2,500)	975.6 (178–3,364)
10 > CD4/CD8 ≥ 2.8 (%)	0/23 (0)	3/38 (7.9)	0/12 (0)	1/25 (4.0)	6/38 (15.8)	2/16 (12.5)	1/13 (7.7)	8/49 (16.3)	21/214 (9.8)
CD4/CD8 ≥ 10 (%)^††^	0/23 (0)	0/38 (0)	0/12 (0)	1/25 (4.0)	28/38 (73.7)	1/16 (6.2)	1/13 (7.7)	2/49 (4.1)	33/214 (15.4)
CD4 + CD7- ≥ 40% (%)^‡^	1/11 (9.1)	4/17 (23.5)	3/11 (27.3)	1/7 (14.3)	12/24 (50.0)	0/7 (0)	2/9 (22.2)	4/24 (16.7)	27/110 (24.5)
CD4 + CD26- ≥ 30% (%)^††^	0/10 (0)	2/25 (8.0)	4/10 (40.0)	2/12 (16.7)	28/28 (100)	0/11 (0)	0/8 (0)	6/42 (14.3)	42/146 (28.8)
Increased LDH (%)*	12/48 (25.0)	40/57 (70.2)	19/19 (100)	24/33 (72.7)	29/38 (76.3)	10/17 (58.8)	8/18 (44.4)	39/51 (76.5)	181/281 (64.4)
Hypoalbuminemia (%)	16/43 (37.2)	16/51 (31.4)	2/19 (10.5)	13/33 (39.4)	8/31 (25.8)	5/15 (33.3)	10/19 (52.6)	23/50 (46.0)	93/261 (35.6)

*P ≤ 0.001; **P = 0.004; ^**†**^*P* = 0.0002; ^**††**^*P* = 0.0001; ^**‡**^*P* = 0.0285.

DL: dermatopathic lymphadenopathy.

N2/3: lymph node involvement by neoplastic cells according to the staging of MF/SS^4^.

### Molecular findings

The TCR gene rearrangement analysis in the skin, blood, and lymph nodes is described in Table 6. In the skin, monoclonality was detected in 39/165 patients (23.6%), was negative in 123/165 (74.5%), and was inconclusive in 3/165 (1.8%). The positivity was higher in SS (22/30, 73.3%), and MF (3/9, 33.3%) (*P* < 0.001). In the blood, monoclonality was practically only observed in SS (90.0%, 30/33) and MF (33.3%, 5/15). In the lymph nodes, monoclonality was detected only in SS (15/17, 88.2%), and MF (2/4, 50.5%) (*P* < 0.001).

**Table 6:** T-cell clonality status on the skin, blood, and lymph nodes.

		Skin	Blood	Lymph node
Psoriasis	Positive (%)	0	0	ND
	Negative (%)	10 (90.9)	10 (100)	ND
	Inconclusive (%)	1 (9.1)	0	ND
Eczema	Positive (%)	3 (9.1)	2 (8.3)	0
	Negative (%)	30 (90.9)	21 (87.5)	5 (100)
	Inconclusive (%)	0	1 (4.2)	0
AD	Positive (%)	1 (10.0)	0	0
	Negative (%)	9 (90.0)	7 (87.5)	1 (100)
	Inconclusive (%)	0	1 (12.5)	0
Drug eruption	Positive (%)	1 (6.2)	1 (11.1)	ND
	Negative (%)	15 (93.7)	8 (88.9)	ND
	Inconclusive (%)	0 (0)	0	ND
SS	Positive (%)	22 (73.3)*	30 (90.9)*	15 (88.2)*
	Negative (%)	7 (23.3)	3 (9.1)	2 (11.8)
	Inconclusive (%)	1 (3.3)	0	0
MF	Positive (%)	6 (50.0)*	5 (33.3)	2 (50.0)*
	Negative (%)	6 (50.0)	8 (53.3)	2 (50.0)
	Inconclusive (%)	0	2 (13.3)	0
Miscellaneous**	Positive (%)	3 (33.3)	2 (20.0)	0
	Negative (%)	5 (55.6)	8 (80.0)	1 (100)
	Inconclusive (%)	1 (11.1)	0	0
Idiopathic	Positive (%)	3 (6.8)	5 (11.9)	0
	Negative (%)	41 (93.2)	36 (85.7)	8 (100)
	Inconclusive (%)	0	1 (2.4)	0
Total	Positive (%)	39 (23.6)	45 (29.8)	17 (47.2)
	Negative (%)	123 (74.5)	101 (66.9)	19 (52.8)
	Inconclusive (%)	3 (1.8)	5 (3.3)	0

*P < 0.001.

** ATLL: positive clonality on the skin 2 (66.7%); positive clonality on the blood 1 (50%).

ND: not done.

### Follow-up

During follow-up (median follow-up 18.5 months; IQR: 6.5–54 months), 28/309 patients (9.1%) died. Sepsis was the most frequent cause of death (25/28, 89.3%), followed by cardiovascular events (3/28, 10.7%). Sézary syndrome and MF groups had the highest mortality rates, with 13/38 (34.2%) and 5/17 (29.4%) deaths, respectively (*P* < 0.001). In 67 cases (21.7%), complete remission was observed, and it was more frequent in drug-induced erythroderma (29/38, 76.3%) and eczema (22/64, 34.4%) (*P* < 0.001). The other 214 patients (69.3%) were alive with evidence of active disease at the last follow-up (Table 7).

**Table 7:** Survival status.

Diagnosis	Alive with disease	Alive without disease	Dead with disease
Psoriasis	45 (86.5%)	4 (7.7%)	3 (5.8%)
Eczema*	42 (65.6%)	22 (34.4%)	0
AD	25 (92.6%)	1 (3.7%)	1 (3.7%)
Drug-induced*	8 (21.0%)	29 (76.3%)	1 (2.6%)
SS**	22 (57.9%)	3 (7.9%)	13 (34.2%)
MF**	12 (70.6%)	0	5 (29.4%)
Miscellaneous	17 (80.9%)	3 (14.3%)	1 (4.8%)
Idiopathic	43 (82.7%)	5 (9.6%)	4 (7.7%)
Total	214 (69.3%)	67 (21.7%)	28 (9.1%)

*P < 0.001: higher remission rates in eczema and drug-induced erythroderma.

**P < 0.001: higher mortality rates in MF and SS.

Six patients with an initial diagnosis of idiopathic erythroderma had the etiology diagnosed during follow-up: two had a diagnosis of eczema, two of MF, one of SS, and one of paraneoplastic erythroderma (lung adenocarcinoma).

## Discussion

The annual incidence of erythroderma varies between 0.9/100,000 persons in the Netherlands^10^ and 1–2/100,000 persons in Finland^11^. Hospital incidence varies from 4.9 cases/year in Thailand^12^ to 35/100,000 dermatological patients in India^13^ and 30–44/100,000 in Tunisia^14^. Brazilian cohorts report a frequency of 1.0–4.7% of inpatients in a dermatology unit with erythroderma^15,16^.

In our study, the median age at diagnosis of erythroderma and the male-to-female ratio are in accordance with the literature (Table 8). An important characteristic of the AD group in our study was the younger age at diagnosis. Cesar *et al*. reported younger age at diagnosis in erythrodermic eczema (mean 46.0 years) and older age in malignancy-associated erythroderma (mean 69.4 years) (*P* = 0.02)^17^. The authors grouped AD with contact eczema, and that may be a reason for the lower age in this group.

**Table 8:** Other studies.

Authors, year	Number of patients	Male: Female	Median age at diagnosis (years)	Pre-existing dermatoses (%)	Drug reaction (%)	CTCL (%)	Paraneoplastic (%)	Miscellaneous (%)	Idiopathic (%)
Nicolis and Helwig (1973)^70^	135	11.3:1	49	27	40	8	3	10	12
Hasan and Jansen (1983)^11^	50	1.94:1	61	54	10	4	0	0	32
King *et al*. (1986)^71^	82			31	34	18	0	1	16
Sehgal *et al*. (1986)^13^	80	2.6:1	41.9	58	20	0	0	0	22
Thestrhup-Pedersen *et al*. (1988)^29^	204	2.24:1	32–69	38.2	25	0	5	16	19
Wilson *et al*. (1993)^30^	50	2.3:1	55	48	8	4	0	4	38
Botella-Estradas *et al*. (1994)^26^	56	4:1	57	66	12.5	12.5	0	0	9
Vasconcellos *et al*. (1995)^31^	247	1,9:1		59	7.3	4.1	0	0.4	29.2
Sigurdsson *et al*. (1996)^32^	102	2.6:1	61	53	5	13	2	1	26
Pal and Haroon (1998)^42^	90	2.8:1	41.6	74	5.5	5.5	0	0	14.6
Leenutaphong *et al*. (1999)^12^	49	2:1	51.7	26.5	38.7	0	2.04	0	32.65
Morar *et al*. (1999)^57^	138	1.9:1	34.7	64.7	22.5	2.2	0	0	10
El Euch *et al*. (2003)^33^	94	1.54:1	39	66	13	11.5	0	1	8.5
Akhyani *et al*. (2005)^34^	97	1.85:1	46.2	57.9	21.6	10.3	0	0	7.2
Rym *et al*. (2005)^35^	80	2.2:1	53.78	67.5	11.25	8.75	0	5	7.5
Kondo *et al*. (2006)^72^	58	1.76	56.89	56.89	18.97	0	0	0	24.14
Fernandes et al. (2008)^15^	170	1.2:1	53.5	58.23	21.77	10.58	0	0.4	9.4
Khaled et al. (2009)^14^	82	1:1	55.13	43.9	21.9	4.87	0	0	25.6
Yuan et al. (2010)^36^	82	4.7:1	53.4	72	17	3.7	1.2	0	6.1
Li et al. (2012)^28^	260	3:1	52.57	67.31	12.69	1.15	0.77	3.85	14.23
César et al. (2016)^17^	103	1.5:1	54.4	65.0	18.4	11.6	0	1	3.9
Present study	309	2.2:1	57	46.2	12.3	17.8	1.3	6.8	16.8

In psoriasis vulgaris, the incidence is higher and more severe in males^18,19^. In AD, there is a slight predominance in females^20,21^; in CTCL, the male-to-female ratio is between 2 and 3^22^; and drug eruptions are more common in women^23^. In our study, erythroderma was more frequent in men in all categories. Whether this difference is due to skin structure and physiology, the effect of sex hormones, socio-cultural behavior, or environmental factors is yet to be known^24^.

The onset of erythroderma is usually gradual and insidious, except in drug-induced cases, where it is typically sudden, and the resolution is faster than the other causes^14,25–27^. It was reported time from the start of the lesions to the diagnosis of erythroderma from a few days to 11 years, and longer duration in psoriasis and lymphomas^14,17,28^. In our study, the median time from the onset of erythroderma to the first evaluation was 4.0 months. It was lower in drug-induced erythroderma and AD, with a median time of 1–1.5 months (*P* = 0.0001). In AD, erythroderma is frequently associated with the suspension of systemic steroids, which can explain the more acute onset.

### Etiology

Similar to our study, the exacerbation of pre-existing dermatoses is the most prevalent cause of erythroderma in the literature, especially eczema and psoriasis (Table 8). Almost 40% of our patients had a history of previous skin disease. It denotes the importance of a detailed anamnesis. Although 22.2% of our patients with erythrodermic psoriasis did not report previous skin disease, it is possible that mild psoriatic lesions were already present and were not noticed or were not correctly diagnosed by the general practitioner. In our experience, erythrodermic psoriasis is commonly observed as a rebound after suspension of systemic steroids, and as a paradoxical effect of biologic therapy. Contrary to most studies^17^, we separated AD from eczema because AD is a very particular group concerning the clinical findings and immunologic mechanisms. All patients with erythrodermic AD had a previous history of typical AD lesions.

The second most frequent cause of erythroderma is drug eruption. Drugs implicated in erythroderma described in the literature are anticonvulsants (especially carbamazepine), penicillin, allopurinol, trimethoprim-sulfamethoxazole, cephalosporins, rifampicin, amoxicillin, sulfanilamide, aminopyrine, indomethacin, and Chinese herbal medicines^1,11,14,26,28–36^. In our study, anticonvulsants were also the most frequently implicated drug (61.9%), especially carbamazepine. Moreover, anticonvulsants and allopurinol are frequently reported as the triggering factor in drug-induced reaction with systemic symptoms (DRESS)^37^. This eruption may evolve to erythroderma, but it shows a peculiar edematous erythroderma, and the search for DRESS criteria is imperative. The definition of the causative drug is frequently difficult since most patients usually take many medications concomitantly. We identified the correct drug, excluding erythroderma caused by anticonvulsants, in only 8/38 cases (21.1%). Thus, diagnosis is highly dependent on a detailed anamnesis searching for the chronology of the eruption, history of previous drug reaction, and frequency of skin lesions with the suspected drug. Suspension of the suspected drug and improvement of erythroderma is the main criteria to confirm the diagnosis^15^. Possibly, some patients with idiopathic erythroderma may have started skin eruption due to a non-identified drug, and despite a posterior suspension of the drug, a chronic state of skin reaction arose.

The frequency of CTCL corresponds to 0 to 18% of the patients with erythroderma (most with 4–5%), and we observed an increased frequency of erythrodermic CTCL (17.8%) in comparison to the majority of the studies (Table 8). Our institution is a tertiary reference center for CTCL, and the improvement of diagnostic tools (flow cytometry, immunohistochemistry, molecular analyses) may facilitate the diagnosis of patients who previously would be classified as idiopathic erythroderma^4,38^.

The group of miscellaneous etiologies is very heterogeneous but have very distinct clinic-laboratory features, making it easier to reach the correct etiology, but difficult to compare with the other well defined erythrodermic disorders. Pemphigus foliaceous was the most common diagnosis (7/21; 33.3%), probably because Brazil is endemic for this disease^39^. Rarely described in the literature^36^, we diagnosed four patients (4/21; 19.0%) with paraneoplastic erythroderma (lung adenocarcinoma, neuroendocrine lung tumor, prostate adenocarcinoma, colon adenocarcinoma), and erythroderma cleared after treatment of the underlying neoplasm. ATLL is an HTLV-1-associated malignancy with peculiar hematologic findings (marked lymphocytosis) and a dismal prognosis^40^. Skin lesions in ATLL are heterogeneous, and erythroderma may be present in 4.2% of the patients^40^. One patient had amyotrophy of interosseous muscles of the hands and hypoesthetic/anesthetic areas, and leprosy was confirmed with the evidence of intact grouped bacilli on skin biopsies^41^. Pityriasis rubra pilaris and seborrheic dermatitis are frequently cited in the literature as causes of erythroderma but were rarely observed in our study^42^. The differences in the frequency of diseases causing erythroderma among different studies may be due to genetic, geographic, and socio-cultural peculiarities.

In the literature, idiopathic cases vary between 7.2% to 38%^14^. We observed 16.8% of patients with idiopathic erythroderma. A close follow-up is essential since some authors suggest that they may represent a pre-SS or a not yet diagnosable erythrodermic CTCL^14,29,36^.

## Clinical findings

### Skin and appendages

The frequencies of diffuse non-scarring alopecia, palmoplantar fissures, lower limb edema, and mucosal lesions in our patients were described, but no consistent association between these findings and the etiologies of erythroderma has been defined. In the literature, palmoplantar keratoderma and nail alterations are more commonly described in psoriasis^17,28^. We observed increased frequencies of palmoplantar keratoderma and onychodystrophy in psoriasis and SS. In non-erythrodermic psoriasis vulgaris, nail alterations are described in up to 50% of the patients^43^. We observed a higher frequency of nail alterations in erythrodermic psoriasis (83.3%), and they include subungual hyperkeratosis, pitting, onycholysis, and oil drop sign. Damasco *et al*. reported paronychia, leukonychia, and onycholysis as the most common nail manifestations in SS^44^. We observed subungual hyperkeratosis, onycholysis, and shiny nails from scratching the skin due to severe pruritus.

Li *et al*. observed areas of healthy skin in 13.46% of the patients, especially in PRP, psoriasis, and eczema^28^. Pal and Haroon observed islands of healthy skin in pemphigus foliaceous, drug-induced erythroderma, and idiopathic cases^1^. In our study, areas of healthy skin were frequently observed in eczema and AD, especially in the central area of the face. We believe that this topography has a yet unknown immunologic peculiarity, especially in AD patients.

We observed vitiligo-like lesions in 17.8% of the patients, with no differences between the etiologies. This finding is rarely described in the literature. Schwartz and Trotter reported the case of an erythrodermic patient with prostate adenocarcinoma who developed achromic vitiligo-like lesions^45^. In classical vitiligo, increased CD8 + cytotoxic T-cell response, together with a decrease in CD4 + T-cells, causes melanocytes destruction^46^. Vitiligo-like lesions have also been described in CTCL and, despite a predominance of CD4 + neoplastic cells infiltrating the skin, a CD8 + subpopulation is responsible for the achromic lesions^47^. If CD8 + cytotoxic response against melanocytes causes achromic lesions in erythrodermic patients and how this response is elicited are matters of further studies.

Rym *et al*. described ectropion on 17.5% of erythrodermic patients^35^. We observed this alteration on 28.7%, suggesting that it is a relatively common alteration, and it is observed even in acute onset erythroderma (*e.g*., drug-induced erythroderma). The presence of ectropion must be carefully evaluated. Ophthalmologic complications may arise if it is not promptly recognized and correctly managed^48^.

### Systemic signs and symptoms

Pruritus is present in almost all patients. Other studies also report the elevated frequency of pruritus, affecting generally more than 90% of the patients^17,28,36^. Interestingly, Khaled *et al*. reported a lower frequency of pruritus (51.6%), and it affected more frequently patients with erythrodermic psoriasis (*P* = 0.0001) and eczema (*P* = 0.03)^14^. Weight loss is frequently associated with lymphoproliferative disorders, but it was found in approximately half of our patients, with no association with CTCL. Weight loss occurs probably due to the intense catabolic metabolism of erythrodermic patients, especially in older patients^2,49^.

Fever was observed in only 6.7% of our patients, but close monitoring of vital signs of erythrodermic patients is essential to prevent severe infectious complications since skin barrier is significantly impaired^50,51^.

Cesar *et al*. associated lymph node enlargement, hepatomegaly, and splenomegaly with lymphoproliferative diseases^17^. We did not observe this association. In patients with inflammatory erythroderma, as well as in most cutaneous T-cell lymphomas, lymph node biopsies showed dermatopathic lymphadenopathy, and only a minority of CTCL patients presented infiltration by neoplastic cells. Thus, lymphadenopathy in erythrodermic patients occurs probably due to generalized skin inflammation. Hepatomegaly and splenomegaly were rarely observed.

### Histopathology

Studies report that 55–66% of skin biopsies contribute to the final diagnosis of erythroderma^14,17,28^. The accuracy of skin histopathology ranges from 48–83%^3,13,52^. As a useful tool for the diagnosis of the etiology of erythroderma, histopathology should be carried out as soon as possible and should be repeated in different areas and different stages of erythroderma^28,36^. We observed a contribution of skin biopsies in 72.4% (199/275). This higher biopsy accuracy may be explained by advances in immune-histochemistry techniques, which allow a better evaluation of the skin infiltrates, especially in CTCL. Spongiosis was frequently observed, not only in eczematous disorders, probably due to topical treatments that contribute to irritation of the damaged skin. Exocytosis of lymphocytes, frequently described in CTCL, was more frequent in SS and MF, but drug eruption and idiopathic erythroderma also showed exocytosis. Non-specific skin histology was observed in 26.3% of SS patients, similar to the 33.3% described in the literature^53^.

Lymph node biopsies show dermatopathic lymphadenopathy in most cases, even in CTCL^17,28,32,36^. In our study, lymph node biopsy was performed in 19.9% of the patients, and infiltration of neoplastic cells was observed in 44.4% of the patients with MF and 28.6% of the patients with SS with no statistically significant difference (*P* = 0.384). All other patients had dermatopathic lymphadenopathy. Erythrodermic MF and SS are aggressive disorders (5-year survival of 55.7 and 48.3%, respectively), but frequently the lymph node enlargement occurs due to inflammation of the skin, and not always due to infiltration by neoplastic cells^54^.

Bone marrow biopsy is rarely performed. Li *et al*. reported bone marrow biopsy in 11 patients: eosinophilia was observed in 5 (3 atopic/eczema, one dermatomyositis, one hypereosinophilic syndrome), normal bone marrow in 3 (drug reaction, AD/eczema, psoriasis), two had hyperplastic bone marrow (one AD/eczema, one psoriasis), and one had alterations due to sepsis (psoriasis with sepsis due to *S. aureus*)^28^. We performed bone marrow biopsy in eight patients. Infiltration by lymphoma cells was observed in two patients with SS and two with ATLL; all other patients had normal bone marrow tissues.

### Imaging

Imaging studies in erythrodermic patients are rarely described. The most frequent alteration we observed was peripheral or central lymph node enlargement, present in 56.2% of the patients, and it was more frequent in SS (75.8%), MF (86.7%), and drug eruption (71.4%). Central lymph node enlargement was observed in all etiologies and was not associated with CTCL. Central lymph node enlargement occurs probably due to the intense and diffuse skin inflammation, similar to what is observed in peripheral lymph node enlargement. Since the biopsy of mediastinal and intraabdominal lymph nodes is technically difficult, it was not performed. Computed tomography helped to find the etiology in two cases of paraneoplastic erythroderma due to lung cancer. No significant alterations were observed in other organs.

### Laboratory findings

Leukocytosis is common in erythrodermic patients (36.0 to 54.9%), and eosinophilia is also frequently observed (20.7 to 26.0%)^14,17,35,36^. Khaled *et al*. associated hypereosinophilia with drug-induced erythroderma and erythrodermic psoriasis^14^. Cesar *et al*. observed a higher frequency of eosinophilia in malignant erythroderma (76.9%) than in benign inflammatory erythroderma (39.8%) (*P* = 0.03)^17^. In our study, as expected, leukocytosis (≥11,000/mm^3^) and lymphocytosis (≥3,400/mm^3^) were associated with SS, but eosinophilia (≥500/mm^3^) was higher in AD and idiopathic erythroderma. Also, IgE was elevated in most patients (84.1%), but the values were much higher in AD. These findings highlight a possible shift to a Th2 immune response in erythrodermic patients from all etiologies.

The sensitivity of search for morphologically atypical Sézary cells is limited by inter-observer subjectivity. Also, Sézary-like cells may be observed in benign inflammatory conditions^55^. We observed Sézary cells in 32 patients, 10 (31.2%) with inflammatory erythroderma. Manual Sézary cell count is progressively being substituted by flow cytometry, since the manual count is very subjective, and immunophenotypic alterations occur earlier than morphologic changes^5^.

We observed increased LDH in 64.4% of the patients, with lower frequency in psoriasis (25%). Elevated LDH is a prognostic marker in cutaneous lymphomas^22^. As expected, increased LDH values were common in erythrodermic CTCL (76.3% in SS and 58.8% in MF), which are aggressive forms of CTCL^22^.

Hypoalbuminemia is frequently described in the erythrodermic case series, and it occurs probably due to extensive protein loss by a high epidermal turnover rate^28,56^. It was observed in 35.6% of our patients, but this reduced albumin levels did not have a significant impact on clinical picture nor at complications indexes of our patients.

HIV infection may exacerbate psoriasis and cause erythroderma, and generalized skin eruption may also be caused by anti-viral drugs used in HIV^57,58^. HTLV-1 infection is the cause of ATLL, and an erythrodermic variant of ATLL have already been described^40,59,60^. We observed HIV infection in only 2.2% and HTLV-1 infection in 2.9%. None of the HIV positive patients had drug-induced erythroderma, but three of the six seropositive patients (50.0%) had psoriasis, suggesting that exacerbation of psoriasis was triggered by HIV infection. Three patients with HTLV-1 infection had ATLL with neoplastic cells on the skin and peripheral blood, but four patients did not have evidence of ATLL at first evaluation. These cases should be closely monitored since ATLL may develop after decades of infection^40^.

### Immunophenotyping findings

The diagnostic criteria for SS include the presence of a monoclonal TCR gene rearrangement on the blood and the skin, more than 1,000 Sézary cells/μL on peripheral blood, and immunophenotypic abnormalities (CD4/CD8 ≥ 10, CD4 + CD7- ≥ 40%, and CD4 + CD26- ≥ 30%)^4^. The combination of these criteria enhances the diagnostic accuracy of SS^61^. One patient with SS did not fulfill these criteria at first evaluation, but subsequent analyses showed significant alterations. Thus, the follow-up of idiopathic erythrodermic patients is essential to reach the correct etiologic diagnosis. On the other hand, 27 patients with inflammatory erythroderma had isolated phenotypic alterations. The severe systemic inflammatory condition of erythrodermic patients may lead to the proliferation of non-malignant abnormal lymphocytes; thus, a complete work-up is essential to differentiate SS from benign inflammatory erythroderma.

### Clonality findings

Monoclonality on the skin has been reported in benign skin conditions including lichen planopilaris, pigmentary purpuric dermatosis, pityriasis lichenoides chronica, pityriasis lichenoides et varioliformis acuta, lupus erythematosus, borreliosis, herpes virus infection, leishmaniasis, and bite reactions^62^. Also, pseudolymphoma may exhibit a T-cell clone on the skin in 25% of the samples^63^. On the other hand, Kubica *et al*. reported that 77.5% of SS patients have a monoclonal population on skin biopsies. These findings are in accordance with our study, with positivity in 73.3% of SS, and 7.0% of benign inflammatory erythroderma with a positive clone on the skin. Monoclonality was observed in only 50.0% of MF cases, and this low positivity could be explained by the small number of neoplastic cells infiltrating the skin in the erythrodermic phase of MF^64^.

Monoclonality on peripheral blood is reported in 86.7% of the patients with SS^65^, and 24% of patients with benign inflammatory erythroderma^66^. Also, a circulating dominant clone may be detected in healthy elderly patients^67^. We observed a positive clone in peripheral blood in 90.9% of SS patients, and 9.1% of the patients with benign inflammatory erythroderma. In the inflammatory cases, the clonal population was detected in patients with 55–77 years, suggesting that aging may be the cause of these positive clones.

### Follow-up

Most studies report favorable prognosis, with few deaths directly associated with erythroderma^13,14,26,28,36^. Death rate was 9.1%. We observed more deaths in SS and MF and more complete remissions in drug eruption and eczema groups. Sézary syndrome and erythrodermic MF are advanced-stage CTCL, with 5-year overall survival rates of 15 to 40% and 40 to 57%, respectively^22^. As expected, withdrawal of the triggering factor leads to the resolution of skin lesions in drug eruption and eczema. Thus, although erythroderma brings much distress and reduces the quality of life, except for erythrodermic CTCL, it does not represent a significant risk to the patient’s life.

Some authors suggest that a close follow-up should be done in idiopathic erythroderma due to the risk of evolving with a CTCL^1,28^. In our study, during follow-up, two patients with idiopathic erythroderma had a diagnosis of eczema, two of MF, one of SS, and one of paraneoplastic erythroderma (lung adenocarcinoma). The other patients remained as idiopathic after a median follow-up of 14.4 months (from 22 days to 8.9 years). These cases highlight the importance of a close follow-up, but the diagnosis of the etiology is difficult, and most patients remain as idiopathic despite extensive and repeated clinical, laboratory, and histopathological reexamination.

These data highlight the importance in performing a detailed anamnesis to search for a possible trigger, and in searching for CTCL criteria, since erythrodermic MF/SS are rare and aggressive diseases, and early referral for treatment in a specialized center is mandatory.

## Conclusion

To our knowledge, this is the largest prospective study of erythrodermic patients. One study limitation is the selection bias since all patients were seen in a tertiary center, but significant conclusions may be drawn. Younger age and high levels of IgE are associated with AD; palmoplantar keratoderma and onychodystrophy are associated with psoriasis and SS. Acute onset of erythroderma are observed in drug-induced erythroderma and psoriasis. Skin biopsy is an essential tool for the diagnosis of the etiology of erythroderma. Immunophenotyping of lymphocytes and the search for T-cell clonality on the blood and the skin are essential for the diagnosis of erythrodermic CTCL. Lymphadenopathy and weight loss are usually associated with lymphoproliferative diseases but were commonly observed in patients with erythroderma irrespective of the etiology, due to the intense chronic inflammatory and catabolic state of these patients.

We suggest that lymph node biopsy should not be performed at the initial evaluation of erythrodermic patients, except in patients with a diagnosis of CTCL, as part of their staging process. In patients with inflammatory or unknown etiology, lymphadenopathy should be observed, and it should decrease as skin inflammation improves. Bone marrow biopsy should be reserved for CTCL patients with laboratory alterations suggestive of infiltration by neoplastic cells. We propose that a CT scan should be performed in patients with CTCL for their staging, but also patients with idiopathic erythroderma, to search for internal malignancies causing paraneoplastic erythroderma. Total IgE dosage should be performed as the initial evaluation of erythroderma because high levels are indicative of AD. We suggest that the diagnosis of erythrodermic CTCL only by the morphology of peripheral lymphocytes is limited to cases with evident lymphocytosis when flow cytometry for immunophenotyping of lymphocytes is not available.

Finally, despite erythroderma do not increases the risk of death (except in CTCL), it imposes significant distress, reducing the quality of life dramatically. Furthermore, standardizing the evaluation of erythrodermic patients is the first step to better define the management and understand the evolution of this severe skin condition.
